# How Can Deep Eutectic Systems Promote Greener Processes in Medicinal Chemistry and Drug Discovery?

**DOI:** 10.3390/ph17020221

**Published:** 2024-02-07

**Authors:** Luis Domingues, Ana Rita C. Duarte, Ana Rita Jesus

**Affiliations:** LAQV-REQUIMTE, Chemistry Department, School of Science and Technology, NOVA University, 2829-516 Caparica, Portugal; lt.domingues@campus.fct.unl.pt (L.D.); ard08968@fct.unl.pt (A.R.C.D.)

**Keywords:** green chemistry, deep eutectic systems, synthesis, pharmaceuticals, drug discovery

## Abstract

Chemists in the medicinal chemistry field are constantly searching for alternatives towards more sustainable and eco-friendly processes for the design and synthesis of drug candidates. The pharmaceutical industry is one of the most polluting industries, having a high E-factor, which is driving the adoption of more sustainable processes not only for new drug candidates, but also in the production of well-established active pharmaceutical ingredients. Deep eutectic systems (DESs) have emerged as a greener alternative to ionic liquids, and their potential to substitute traditional organic solvents in drug discovery has raised interest among scientists. With the use of DESs as alternative solvents, the processes become more attractive in terms of eco-friendliness and recyclability. Furthermore, they might be more effective through making the process simpler, faster, and with maximum efficiency. This review will be focused on the role and application of deep eutectic systems in drug discovery, using biocatalytic processes and traditional organic chemical reactions, as new environmentally benign alternative solvents. Furthermore, herein we also show that DESs, if used in the pharmaceutical industry, may have a significant effect on lowering production costs and decreasing the impact of this industry on the quality of the environment.

## 1. Introduction

The production of active pharmaceutical ingredients (APIs) is responsible for the high amount of waste in the pharmaceutical industry, especially the solvent-related waste. In fact, GSK reported that their APIs production generates up to 80% of their solvent-related waste [1]. If all pharmaceutical companies produce the same amount of waste, this represents a huge impact on the environment. Therefore, there is an urgent need for alternatives to overcome this major problem. The most effective way to change this trend is the replacement of volatile organic solvents with alternative solvents during the synthesis of APIs.

Green chemistry has gained more and more attention from the pharmaceutical industry. While many alternative solvents, such as fluorinated solvents, supercritical fluids, and biomass-derived solvents, have been considered green solvents, many fail to obey green chemistry principles. However, deep eutectic solvents (DESs), on the other hand, comply with most of the green chemistry principles. Herein, the use of DESs as alternative solvents in medicinal chemistry and drug discovery, namely their use in biocatalytic processes and organic synthesis, will be summarized and discussed.

## 2. Deep Eutectic Systems

Deep Eutectic Solvents (DESs), i.e., a mixture of two or more compounds that suffer a significant depression in the melting point of each component, were first reported by Abbott et al. [2]. In 2003, they reported a system composed of choline chloride:urea (1:2), two solids at room temperature, with very high melting points (302 °C and 133 °C, respectively) and observed that the melting point of the mixture was −12 °C, meaning that it is liquid at room temperature, representing a significant decrease in their melting points. DESs are mainly formed through the establishment of intermolecular hydrogen bonds, usually between hydrogen bond donors and hydrogen bond acceptors. In terms of physicochemical properties, DESs have: (i) densities usually higher than water, except for hydrophobic systems, (ii) high viscosities at room temperature, (iii) poor conductivity at room temperature due to high viscosity, (iv) polarities dependent on the constituents, (v) different affinity to aqueous systems, depending on the composition, (vi) non-flammability, (vii) non-volatility, (viii) thermal stability, (ix) low toxicity, and (x) high biodegradability [3].

After Abbott’s first report, DESs have emerged mostly with the intention of not only replacing common volatile organic solvents, such as dichloromethane, methanol, chloroform, etc., in many different applications, but also as an alternative to ionic liquids [4].

When it comes to the preparation and discovery of active pharmaceutical ingredients (APIs), DESs have been reported as performing different roles, namely, as solvents or with a solvent/catalyst dual function. Because of this dual role, DESs will be referred to as deep eutectic systems rather than deep eutectic solvents.

Furthermore, DESs can also be used in biocatalytic transformations with pharmaceutical relevance. These topics will be further discussed in the following sections.

### 2.1. DESs as Alternative Solvents in Biocatalytic Processes in Drug Discovery

Biocatalysis has been defined as a chemical reaction in which enzymes, obtained from biological sources or whole cells, are employed to speed up the reaction [5].

In the pharmaceutical industry, biocatalysis has become crucial for the preparation of several APIs, mainly due to the sustainability and efficiency associated with these types of processes. Biocatalysts have the great advantage of conferring unparalleled selectivity during the synthesis of pharmaceuticals [6]. Furthermore, when optimized it can lead to robustness and scalable reactions. In the early days, hydrolase reactions were largely performed in the pharmaceutical industry [7], but others followed, including keto-reductases, transaminases, aldolases, and hydroxy nitrile lyases. Currently, researchers have been looking for other enzyme classes to be used in chemical processes, bridging the vast number of enzymes present in nature and the limited number of enzymes used in biocatalytic processes.

Although biocatalysis is seen as the future of the pharmaceutical industry, mainly because it requires water and phosphate buffers, thus completely avoiding the use of organic solvents, it presents a major drawback, which is related to the water solubility of raw materials [8]. Alternative solvents are being investigated, but such solvents should be non-toxic, biocompatible, biodegradable, and sustainable. It is also important that it should confer stability and maintain the enzyme activity. It has been reported that enzymes can remain stable and active in non-aqueous solvents, such as glycerol, but this is not applicable to all classes of enzymes [9,10,11]. Other alternatives have been searched, namely the use of deep eutectic systems (DESs) [12,13].

In the literature, it is possible to find several reports using DESs in biocatalysis, namely in reductions, oxidations, hydrolysis, esterification, and transesterifications, using isolated/immobilized enzymes or whole cells [13]. Although most reactions are reported per se, they can potentially be used in the preparation of pharmaceuticals. In the next sections, examples of biocatalytic processes for the preparation of bioactive molecules or pharmaceuticals, using DESs as an alternative reaction medium, will be summarized and discussed.

#### 2.1.1. Enzymatic Reduction of Ketones and Aldehydes to Alcohols

The reduction of ketones and aldehydes into alcohols is quite useful to prepare APIs. At Hoechst AG in Germany, researchers use NaBH_4_ to reduce a ketone group to an alcohol in the preparation of an intermediate of HMG-CoA reductase inhibitors [14]. Another example, at Novartis, is the racemic synthesis of Fluvastatin, including the reduction of a ketone group into a racemic *syn*-1,3-diol through chemical methods [15]. However, Cicco et al. reported the first application of DESs on the asymmetric bioreduction of ketones using purified ketoreductases (KREDs) [16]. The authors showed that choline-based DESs (ChCl:Gly, 1:2 or ChCl:Sor, 1:1) were able to reach >99% conversion and up to a >99% enantiomeric excess of the corresponding alcohol, while in neat buffer, >99% conversion was also achieved but with far less stereoselectivity. In fact, they showed that the increase in the DESs % led to a gradual improvement on the stereoselectivity, reaching >99% ee using 80% (*w*/*vw*) of the DESs.

**Scheme 1:**
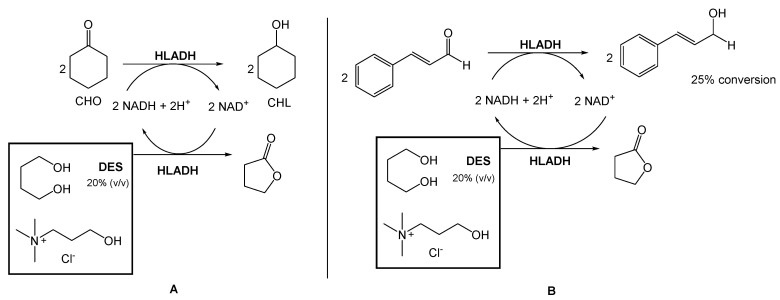
(**A**). Bioreduction of cyclohexanone (CHO) and (**B**) Bioreduction of cinnamaldehyde using choline chloride:1,4-butanediol eutectic system, reported by Chanquia et al. [17,18].

Several authors have been reporting the use of alcohol dehydrogenases (ADHs) from horse liver (HLADH) to reduce ketones and aldehydes to alcohols. However, ADHs are cofactor-dependent, usually being combined with an *in situ* generation system, which requires a second enzymatic step mediated by glucose dehydrogenase or formate dehydrogenase. To overcome this need for a second reaction, Chanquia et al. have been exploring the use of smart co-substrates, which shift the reaction equilibrium, transforming the substrate into a thermodynamically and kinetically stable co-product [17,18]. As a model reaction, reduced cyclohexanone (CHO) to cyclohexanol (CHL), using a buffered solution of choline chloride:1,4-butanediol eutectic mixture as reaction medium (20% *v*/*v*), to decrease the viscosity and to provide optimal enzyme activity (Scheme 1A) was used. They were able to obtain a quantitative conversion of CHO to CHL, after 5 h. Afterwards, they reduced cinnamaldehyde to cinnamyl alcohol using the same conditions, with 25% conversion after 48 h (Scheme 1B).

This conversion is highly relevant from an industrial point of view because the cinnamyl alcohol is an intermediate in the production of flunarizine, a selective calcium entry blocker with calmodulin binding properties and histamine H1 blocking activity, used to treat migraines, occlusive peripheral vascular disease, vertigo of central and peripheral origin, and as an adjuvant in the therapy of epilepsy.

**Scheme 2:**
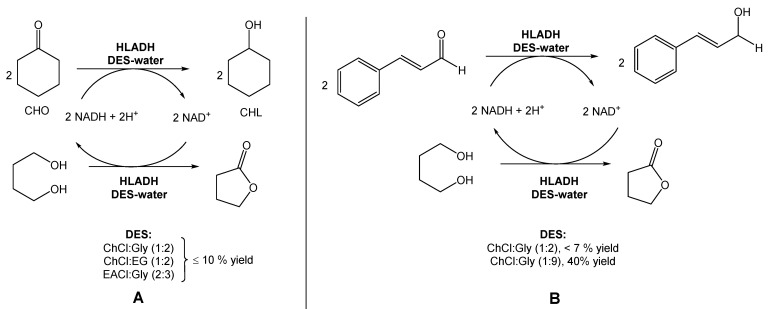
(**A**) Alternative bioreduction of cyclohexanone (CHO) into cyclohexanol (CHL) and (**B**) alternative bioreduction of cinnamaldehyde using ChCl:Gly (1:2 vs. 1:9), reported by Bittner et al. [19].

In a different report, Bittner et al. investigated the use of ChCl:Gly (1:2), ChCl:EG (1:2), and EACl:Gly (2:3) for the reduction of cyclohexanone into cyclohexanol, also using HLADH (Scheme 2A) [19]. In this study, contrarily to Chanquia’s work, the authors added 1,4-butanediol to the DES mixture. Their findings suggested that, in this case, the use of pure glycerol would be the best choice to achieve the maximum reactivity. However, the high viscosity of glycerol limits its applicability in these reactions. Therefore, the use of glycerol-based DESs provided less solvent viscosity and facilitated the manipulation of glycerol–water mixtures. Inspired by these results, the authors used a DES composed of choline chloride and glycerol, but with a higher glycerol content (1:9) to evaluate the reduction of cinnamaldehyde to cinnamyl alcohol (Scheme 2B). The use of this new DES allowed them to reach a 2.5-fold higher specific activity and a 40% conversion, instead of less than 7% when using a DES with a lower content of glycerol (ChCl:Gly (1:2)).

**Scheme 3:**
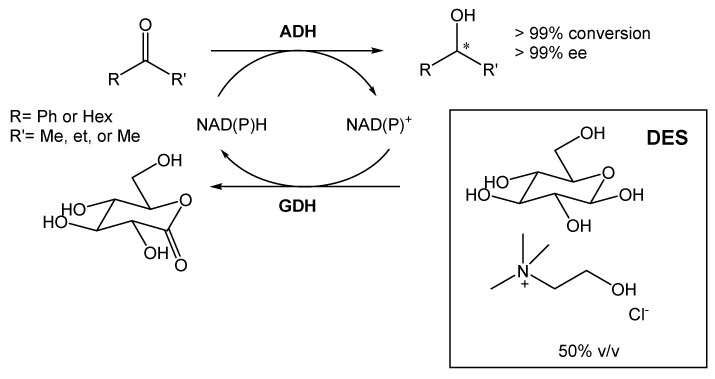
Alcohol dehydrogenase-catalyzed bioreduction of ketone using ChCl:Glu (1.5:1), able to recycle the nicotinamide cofactor [NAD(P)H].

A DES composed of choline chloride and glucose (1.5:1) was also employed for the bioreduction of ketones into alcohols, using different alcohol dehydrogenases (ADHs) (Scheme 3) [20]. The ratio selected between the two components was chosen so that glucose could be used for cofactor recycling. Furthermore, Mourelle-Insua et al. studied the combination of an aqueous buffer with the DES at up to 50% (*v*/*v*) [20]. The use of such high NADES concentration showed two main advantages. First, the presence of glucose enabled the nicotinamide cofactor recycling. Then, the authors were able to conduct the reaction in much higher substrate concentrations in comparison with the buffer system employing glucose/glucose dehydrogenase (GDH). This strategy allowed them to obtain the desired products with >99% conversion and >99% ee.

**Scheme 4:**
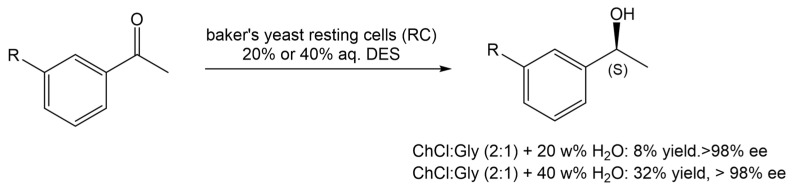
Bioreduction of acetophenone derivatives in the presence of baker’s yeast and an aqueous solution of DES.

In another report, Vitale and co-workers [21] reported the use of ChCl:Gly (1:2) in 20 or 40% water (*w*/*v*), in the stereoselective enzymatic reduction of acetophenone derivatives with baker’s yeast RC, during the preparation of rivastigmine, an API used to manage and treat neurodegenerative diseases, such as dementia, in patients with Alzheimer’s and Parkinson disease (Scheme 4). From the several reaction conditions tested, it was observed that in a 40 w% aqueous solution of ChCl:Gly (2:1), it was possible to achieve the highest reaction yield. When water was used as the medium, the maximum yield was 27% after 120 h of reaction.

The examples presented in this section confirm the applicability of DESs in the biosynthesis of several compounds, relevant intermediates for the preparation of APIs. Furthermore, the results are quite promising, with high conversion rates and a high stereoselectivity, which is sometimes significantly difficult to achieve using traditional synthetic approaches.

#### 2.1.2. Bioreduction of Imines to Amines

About 40% of pharmaceutical drugs contain a chiral amine group. These can be obtained through the hydrogenation of imines, enamines, and their derivatives; but most of these reactions require expensive precious metal catalysts [22]. Because of this limitation, several sustainable biocatalytic routes are being used in the industry for the preparation of chiral amines that include lipases [23], transaminases [24], ammonia lyases [25], amine dehydrogenases [26], and amine oxidases [27]. In 2010, imine reductases (IREDs) were first described for the reduction of the imine 2-methyl-1-pyrroline into (R) or (S)-2-methylpyrrolidine [28]. Since then, interest on this enzyme family has grown [29]. These enzymes are NADPH-dependent enzymes able to successfully produce primary, secondary, and tertiary amines from cyclic imines.

**Scheme 5:**
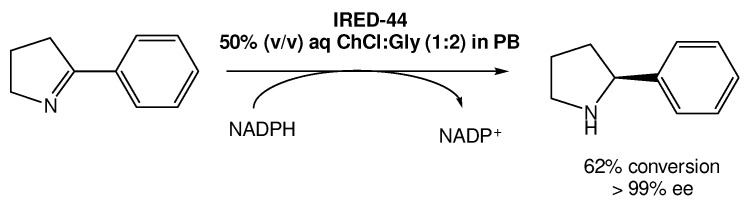
Imine enzymatic reduction in a 50% (*v*/*v*) DES/PB mixture in the presence of IRED-44.

Recently, Arnodo et al. reported the use of IREDs in DESs for the reduction of cyclic imines into amines (Scheme 5) [30]. In this study, they started by using a choline chloride:glycerol DES (1:2) using the IRED-44 enzyme. They observed that when the reaction was carried out in pure DES, which has a lower viscosity when compared with other DESs, it was not possible to recover the desired product, even when using a high concentration of the substrate. Therefore, they attempted to use a 50% (*v*/*v*) DES solution in phosphate buffer (PB), which is the optimal amount of DES used in other studies using other reductase enzymes, such as KREDs. This change in the protocol led to the recovery of the product in good yield in both PB and DES/PB mixtures, even at low concentrations of the substrate.

However, the authors observed that once using higher concentrations (100 mM) of the substrate, the enzymes became ineffective, without product formation. But, using the DES/PB mixtures as solvents they were able to not only recover the product, but also improve the reaction yield, reaching a maximum of 62% yield with >99% ee. Nevertheless, even when using substrate concentrations higher than 100 mM it was possible to reach 49–51% yields, which does not represent a significant decrease in its efficiency.

Overall, another important and relevant outcome from this initial study was that it was possible to work at higher concentrations without the need to increase the solvent volume.

**Scheme 6:**
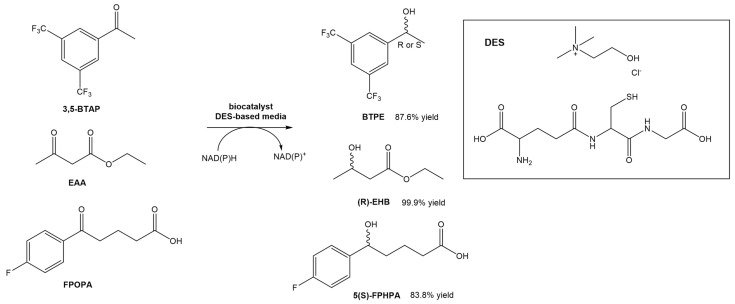
Bioreduction of 3,5-BTAP, EAA and FPOPA in a DES-based medium.

Wang et al. designed a new DES composed of choline chloride and glutathione, and utilized it for the asymmetric reductions of 3,5-bis(trifluorometyl) acetophenone (3,5-BTAP), an important intermediate in the preparation of (R)-[3,5-Bis(trifluoromethyl)phenyl]ethanol ((R)-BTPE), which is a building block in the synthesis of antiemetic drugs used to prevent chemotherapy-induced side effects, such as aprepitant and fosaprepitant (Scheme 6) [31]. In this study, the authors reported the use of whole cells of *Trichoderma asperellum* ZJPH0810. Similarly to other studies using DESs in biocatalytic processes, also in this report the authors were able to significantly increase the concentration of the substrate (up to a 2-fold increase). Comparing the reaction in the absence or presence of the DES in the solvent, the yield increased from 53.1% to 70.8%. The authors even compared the effect of replacing the DES with the [BMIM][PF_6_] ionic liquid. In terms of substrate concentration, the use of a DES-based medium and an IL-based medium allowed the use of higher concentrations (70 mM—IL vs. 100 mM—DES). In terms of conversion yield, the presence of ChCl:GSH afforded higher values (87.6%) than using the [BMIM][PF_6_] ionic liquid (82.5%). Later, they tested the same DES in other conversions, namely the reduction of ethyl acetoacetate (EAA) and 5-(4-fluorophenyl)-5-oxopentanoic acid (FPOPA) (Scheme 6). In the first case, the conversion of EAA in EHB resulted in similar results (83.8% yield and >99 ee%) when the reaction was performed in a DES-based medium and water, although faster. In the second case, the biocatalytic reduction of FPOPA in the presence of the DES resulted in significantly higher reaction yields than in water, after the same reaction time (83.9% vs. 65.9%). Both reactions were carried out with the same substrate concentration and reaction time. In this work, the authors showed that the ChCl:GSH system is a promising cosolvent for bioreductions.

#### 2.1.3. Oxidation

Many bioactive molecules are prepared through oxidated building blocks, hence the high relevance of bio-oxidations. Enzymes employed in this type of reaction have a metal core (either iron or copper) that interacts with oxygen atoms, promoting their transfer to the substrates.

Laccase, a copper-containing enzyme, is one of the most used enzymes in bio-oxidations. It catalyzes the single-electron oxidation of organic and inorganic compounds through the reduction of molecular oxygen in water [32].

To understand the applicability of DESs as solvents or cosolvents in laccase-catalyzed reactions, Toledo et al. investigated the effect of 16 aqueous choline- and betaine-based DES solutions on laccase activity, under different conditions [33]. The preliminary results showed that the presence of ChCl in the media deactivated the enzyme, especially if the DES is used at a high concentration (50 w%). They further confirmed the negative effect of ChCl by increasing the ChCl ratio in DESs (1:2 vs. 2:1), supporting the fact that the Cl ion is responsible for the deactivation of the enzyme, as reported in the literature [34]. Replacing the ChCl with cholinium-based salts was investigated, namely, ChDHP (choline dihydrogen phosphate) and ChDHC (choline dihydrogen citrate), and significant improvements were observed. Regarding the betaine-based DES aqueous solutions they were able to maintain, or even increase by 20%, the laccase activity when compared to the buffer control.

This study showed that DESs should be carefully chosen for a specific reaction to improve biocatalytic performance. Nevertheless, it also showed the potential of these new solvents in laccase-based biocatalytic processes.

**Scheme 7:**
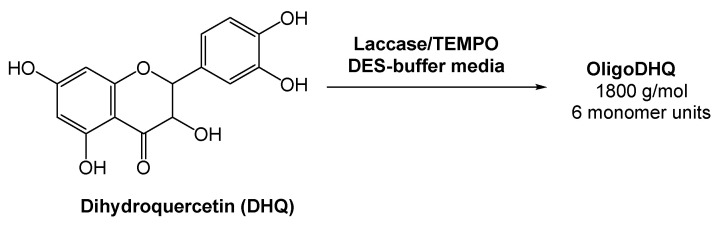
Laccase-catalyzed oxidative polymerization of dihydroquercetin.

Oligomeric derivatives of flavonoids are promising molecules for pharmaceutical applications due to their strong antioxidant, anticarcinogenic, and antiviral properties. Considering this, Yaropolov and co-workers [35] investigated the laccase-catalyzed oxidative polymerization of dihydroquercetin (DHQ), a natural flavonoid, in the presence of a (2,2,6,6-tetramethylpiperidin-1-yl)oxyl radical (TEMPO), and using a DES-derived buffer as the medium (Scheme 7). The authors evaluated the activity of laccase in the pure DES and observed that it resulted in the complete inactivation of the enzyme. But, as previously showed and reported by several authors, the oxidoreductase activity is maintained using DES-buffered solutions [33,36,37,38]. Bearing this in mind, the reaction using a 60% (*v*/*v*) aqueous system Bet:Gly (1:2) and 0.61% of TEMPO was investigated. A high loading of DHQ was reached (>17 mM) and the enzyme remained stable for 12 h. This reaction afforded a product with 58% yield and a molecular weight of 1800 g/mol, with an average length of six monomers. The results showed that the eutectic system Bet:Gly (1:2) can be used for the laccase-catalyzed polymerization of dihydroquercetin, thus fulfilling the green chemistry parameters.

**Scheme 8:**
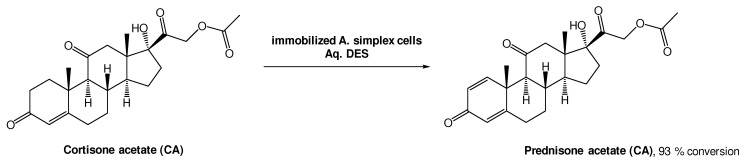
Δ1,2-dehydrogenation bioreaction using Arthrobacter simplex cells in aqueous solutions of DESs.

A different approach, using whole cell oxidations is also common to produce bioactive molecules. Prednisone acetate (PA) is a steroid with anti-inflammatory and anti-allergy properties, and it can be obtained from cortisone acetate (CA) through a Δ1,2-dehydrogenation reaction using *Arthrobacter simplex* cells (Scheme 8). Lu et al. investigated this biotransformation using choline-based DESs as alternative solvents. From the three DESs tested, ChCl:Gly, ChCl:EG, and ChCl:U, the last one showed the best performance, allowing for a 93% conversion [39]. The authors also tested the recyclability of this medium and, interestingly, only a slight decrease to 81% conversion after five cycles, was observed. More importantly, the conversion was more efficient using a DES-based aqueous medium (93%) than without the DES (72%), demonstrating their high relevance in biohydrogenation reactions.

**Scheme 9:**
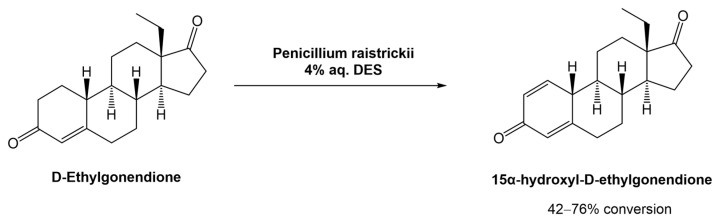
Biocatalytic 15α-hydroxylation of D-ethylgonendione in aqueous solutions of DESs.

In 2020, Mao et al. proposed and studied the combination of the fungus Penicillium raistrickii and DESa in the industrial process for the 15α-hydroxylation of D-ethylgonendione (Scheme 9), which is a key intermediate in the synthesis of gestodene (15α-hydroxyl-D-ethylgonendione), a progestin used in menopausal hormonal therapy [39].

In this study, 16 choline-based DESs were used. It was observed that the composition of the DESs had a great impact on the success of the biotransformation. For example, when ChCl was combined with organic acids (lactic acid, oxalic acid, malonic acid, citric acid, and malic acid), there was no recovery of the desired product, mostly linked to the acidity of the medium which can inactivate the fungus. Comparing the combination of ChCl with sugars (glucose, fructose, sucrose, or xylose), urea, or polyols (ethylene glycol, glycerol, or sorbitol), the results showed that the polyols afforded the product in higher yields (>50%), with ChCl:Gly (1:2) being the best system (76% conversion). Although the ChCl:sugar systems also showed high efficiency in the biotransformation (42–46%), their viscosity hindered the product isolation. The authors compared the efficiency of the DESs against the physical mixture (ChCl + Gly) and the individual components alone in the reaction media, in which the DESs worked significantly better.

The concentration of the DES in the media was also optimized and 4% (*v*/*v*) afforded the best results, which can be justified by the high viscosity when higher concentrations of DESs were used. Furthermore, they also compared the reaction time against conversion rate with and without the DES. The substrate conversion in DES-based media reached 82% after 72 h, while the control reaction, i.e., without DES, was only 33% after the same time. Additionally, the authors showed that the DES ChCl:Gly (1:2) is highly biocompatible with the fungus used in these transformations.

#### 2.1.4. Hydrolysis

Hydrolases, another common enzyme family used in the preparation of relevant molecules, catalyze bond cleavage using water, and the transformation does not require the use of cofactors.

**Scheme 10:**
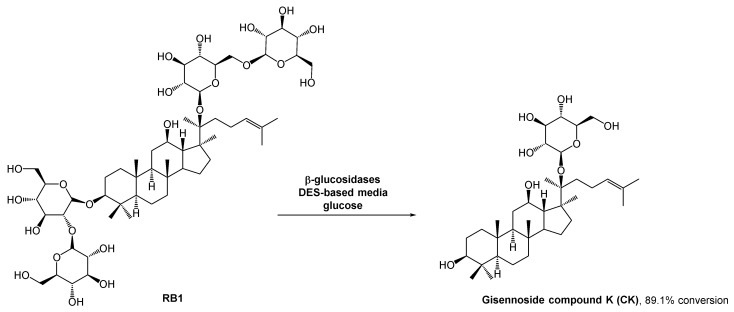
β-Glucosidase catalyzed hydrolysis of RB1 to afford ginennoside compound K (CK) in the presence of a DES-aqueous medium.

The gisennoside compound K (CK) is well absorbed in the body and is the main compound responsible for ginseng’s bioactivity, including antitumoral bioactivity. Since this compound is rarely found in natural sources, researchers have been searching for an easy way to prepare it, and enzymatic strategies have aroused their interest. The methods for CK preparation can be found in the literature [40,41,42]. However, the poor solubility of some intermediates can hinder the outcome. The use of organic solvents could solve this solubility problem, but it is against the green chemistry principles. DESs emerged as a potential solution for solubility and enzyme stability problems, and since there were no enzymatic processes reported for the ginsenoside conversion, Han et al. studied the use of these alternative solvents in CK biosynthesis (Scheme 10) [43]. They combined choline chloride with different hydrogen bond donors such as glycerol, ethylene glycol, and urea. After some studies, they concluded that the performance of the enzymatic reaction was significantly higher in polyols-based DESs. In terms of substrate solubility, the substrate was much more soluble in the DES–aqueous medium than in acetate buffer. The reaction carried out with 30% (*v*/*v*) ChCl:EG (2:1) reached the maximum conversion (89.1%), while in acetate buffer it was only possible to reach 29.1% conversion.

As expected, higher DES concentrations lead to lower yields, resulting from the inactivation of the enzyme. In this study, the authors also observed that when using high concentrations of the substrate, the reaction yield decreased. Therefore, a fed-batch system was developed in which the substrate was continuously fed into the reaction vessel and the product was continuously removed. This strategy mitigated the inhibition caused by the high viscosity of the initial mixture and the accumulation of CK, and might be a sustainable solution for the large production of CK, thus allowing to increase the conversion yield up to 89.1%%, 29% higher than using only traditional aqueous media.

Polyphenols, such as quercetin, have several biological activities, hence they are desired target molecules for both the food and pharmaceutical industries [44,45,46] Most flavonoids are found in flowers, leaves, and fruits, mainly in glycosides and aglycones forms.

**Scheme 11:**
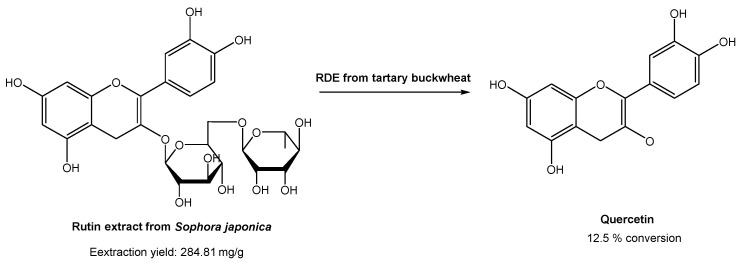
Quercetin preparation from rutin extracted from Sophora japonica, in a DES-aqueous medium, using degrading enzyme (RDE) from germinated tartary buckwheat.

In industrial processes, quercetin is usually prepared via hydrolysis from rutin, which is found in high content in Sophora japonica. Rutin is usually extracted using traditional organic solvents. However, many authors have already reported the use of DESs for the extraction of rutin from Sophora japonica [47,48,49,50,51]. All of them found that DESs are much more efficient for rutin extraction than common organic solvents, such as ethanol and methanol. Based on these studies, Zang and co-workers [52] studied the conversion of rutin, extracted with DESs, in quercetin with the degrading enzyme (RDE) obtained from germinated tartary buckwheat in situ (Scheme 11).

Briefly, several DESs were used to extract rutin, but the most promising ones were ChCl:Gly and EG:Ala. Later, they studied the effect of water in the extraction process. In the case of ChCl, the increase in water content up to 20% increased the rutin recovery efficiency. On the other hand, the increase in water content in EG:Ala resulted in a decrease in the extraction efficiency. After selecting the best system, the activity of degrading enzyme in DESs was evaluated to assess its applicability of rutin conversion into quercetin. Using ChCl:Gly with 20% water, the best catalytic activities were obtained. After the optimization of the catalytic reaction conditions (pH, time, substrate concentration, enzyme amount, and temperature), it was possible to reach only 12.5% conversion. Although the traditional process reached higher yields (50% yield), the methodology proposed by the authors is far more sustainable and environmentally friendly, since the study involved sustainable and eco-friendly solvents for both substrate extraction and catalytic reaction, and even the enzyme was isolated from a plant.

#### 2.1.5. Esterification and Transesterifications

Esterification and transesterification are among the most common organic transformations in industry, including the pharmaceutical industry. Lipases are probably the mostly used enzymes in these transformations [53,54,55,56,57]. In drug discovery, esterification can play an important role, especially in the design of prodrugs, commonly used to improve the pharmacokinetic and pharmacodynamic properties of a drug [58].

For example, dihydromyricetin (DMY), a flavonol derivative showing promising bioactivities that include antioxidant, anti-inflammatory, analgesic, and others. However, the corresponding acetate presents an improved liposolubility and even an improved antioxidant activity. The chemical synthesis of DMY acetate presents several problems, namely low yield, low regioselectivity, and harsh reaction conditions, and it requires time-consuming purification steps. To overcome all these problems, enzymatic synthesis has been considered, but solvent incompatibility also represents an issue. Cao et al. explored the acylation of DMY using *Aspergillus niger* lipase (ANL) which was immobilized in magnetic nanoparticles (ANL@PD-MNPs) using a DES as the reaction medium. They used DESs (ChCl:Gly, ChCl:Xyl, ChCl:U) combined with DMSO, which was already successfully used in these types of reactions [59,60]. The substrate was significantly more soluble (1.73-fold) in DESs than in DMSO alone; however, the reaction did not occur in the pure DES. Therefore, the authors screened different volume fractions of DES/DMSO and concluded that 1:3 (*v*/*v*) was the optimal value, allowing a 91.3% conversion against 18.8% with 3:1 (*v*/*v*). In DMSO alone, the conversion rate is reported as 79.3% [61], hence a significant improvement by the combination of DMSO with DES.

The authors also evaluated the recyclability of ANL@PD-MNPs in ChCl:Gly/DMSO (1:3, *v*/*v*). The enzyme retained its catalytic activity (>90%) up to 5 cycles, although after 10 cycles, its activity still allowed a 56.7% conversion.

In a different study, conducted by Liu and co-workers, 1-caffeoylglycerol (1-CG) was obtained via a transesterification from methyl caffeate (MC) and glycerin, using Novozym 435 in several solvents, including DMSO, acetone, and DES media. Using a batch reactor, the maximum reaction yield, 90.63%, was obtained using 10% (*v*/*v*) ChCl:U in glycerol, at 75 °C and 10 h. As an alternative, using a microreactor at 65 °C for 150 min, the reaction yield was significantly improved, up to 96.44%. The authors observed that the temperature played a significant role in this high yield, most likely due to the drastic decrease in the viscosity of the reaction media.

The recyclability of Novozym 435 was also evaluated, due to its relevance for industrial applications. A significant decrease in reaction yield was observed after only 16 cycles, although after 20 runs the yield remained higher than 50%, showing that when the reaction is carried out in the microreactor, the catalytic activity and regioselectivity of the enzyme is maintained.

Although some examples use DESs for the esterification and transesterification of biomolecules, the majority of examples on the use of DESs in lipase-catalyzed reactions is mostly for the modifications of edible oils or for racemization processes [62,63,64,65,66]. Since these transformations are not within the scope of this work they will not be discussed herein.

#### 2.1.6. Other Transformations

The amine and amide chemical groups are widely spread among active compounds, including peptides and oligo peptides.

Cefaclor, a cephalosporin second-generation antibiotic, has been prepared using biocatalysis through the immobilization of penicillin acylase in magnetic nanocrystalline cellulose [67]. But the substrate 7-amino-3-deacetoxycephalosporanic acid (7-ACCA) presented a low solubility in aqueous buffer, limiting the reaction rate and leading to undesirable by-products. The use of traditional organic solvents, such as DMSO, ethanol, acetonitrile, acetone, etc., could easily solve this solubility issue. However, their incompatibility with enzymatic systems limits further development.

**Scheme 12:**
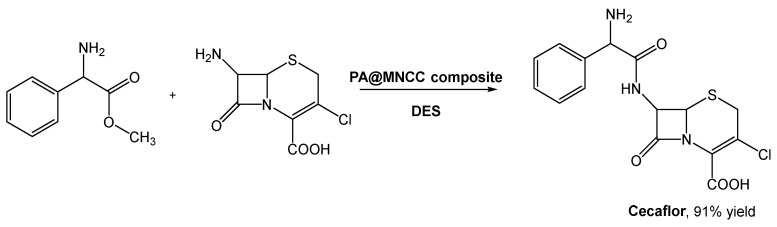
Biosynthesis of Cecaflor using DESa as solvents and penicillin acylate in immobilized magnetic nanocrystalline cellulose (PA@MNCC).

As mentioned before, DESs emerged to solve problems like this, and Wu et al. investigated the preparation of cefaclor using penicillin acylate (PA) in immobilized magnetic nanocrystalline cellulose (MNCC) and DESs as reaction media (Scheme 12) [68]. They combined choline chloride with compounds such as organic acids (citric acid, malic acid, oxalic acid, p-toluene sulfonic acid, and tartaric acid), polyols (butyl glycol, EG, glycerol, propylene glycol, and xylitol), and others (urea and imidazole). When the reaction was screened using the selected DES, ChCl:EG (1:2) in aqueous buffer (30% *v*/*v*) afforded the highest yield (ca. 89%), slightly higher than in water. Although this was already a good result, the authors noticed that the increase in DES:buffer lead to the improvement of the reaction outcome, reaching the maximum yield (91.2%) at 70% (*v*/*v*) concentration of DES. Considering the side reaction, namely hydrolysis, the authors verified that it was possible to increase the synthesis to hydrolysis (S/H) ratio from 1.24, in water, to 1.8 in DES–buffer mixtures.

In this section, we have summarized the application of DESs in biocatalysis used to produce either intermediates for the preparation of APIs, or the APIs themselves. These show the great potential of DESs in biocatalytic processes in the pharmaceutical industry. From a green chemistry point of view, the design of tailored solvents for biocatalytic reactions can contribute to improved synthetic strategies. These alternative solvents facilitate the solubilization of poorly water soluble reactants and confer the stability and enhanced bioactivity of the catalyst.

### 2.2. DESs as Alternative Solvents in Organic Synthesis in Drug Discovery

Although biocatalytic processes are gaining the attention of pharmaceutical companies, traditional organic synthesis is still used in the production of most APIs. In these, the role of the solvent is crucial to achieve a good outcome. Solvents can play a dual role: as solvents, but also as catalysts or reducing agents. Traditional solvents, such as methanol, dimethyl sulfoxide, acetone, dimethyl formamide, and others, struggle to meet the sustainability required nowadays in the pharmaceutical industry [69]. Alternative solvents, including deep eutectic systems, emerged to solve this problem. Several reports in the literature can be found using DESs as alternative solvents for the synthesis of bioactive molecules and pharmaceuticals; however, the use of DESs has not yet been implemented at the industry level. This is mostly because there is still reluctance and a lack of studies in using these alternative solvents on larger scales. Furthermore, currently used industrial processes are well established and changing them without significant proof of benefits would cost companies a huge amount of money. However, this change also represents a high risk/high gain change. For this reason, studies at a larger scale are an urgent need, so that DESs can be implemented at an industrial level.

In the next sections, several examples of organic reactions, usually performed in the preparation of APIs, will be reviewed, using DESs as solvents.

#### 2.2.1. Carbonyl–Olefin Metathesis

Olefin–olefin metathesis has been widely studied and explored in the synthesis of relevant molecules [70]. However, carbonyl–olefin metathesis, also known as alkyne–carbonyl metathesis (ACM) is far less explored, as the methods require photochemical conditions, stoichiometric amounts of transition-metal reagents, or other specific conditions; but recently, this reaction has regained the attention of researchers, because carbon–carbon formation is fundamental in the synthesis of biologically active molecules such as pharmaceuticals and natural products [71].

2H-Chromene, for example, is a structural building block for the synthesis of bioactive molecules, and some of them can be found in natural sources like tannins and polyphenols [72,73]. An array of derivatives found in the literature exhibit interesting activity, namely, antioxidant, anticancer, antitumor, anticoagulant, fungicidal, and anti-HIV activity properties [72,74]. Furthermore, the photochromic properties of some of these derivatives allows for the study of their function as fluorescence probes, and their overall light emission function in materials [75].

**Scheme 13:**
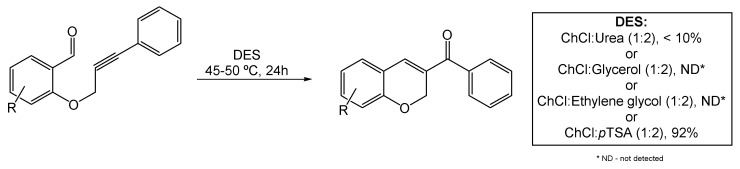
Alkyne–carbonyl metathesis reaction for the synthesis of 2Hchromene derivatives in DESs as alternative solvents.

Recently, Annes et al. explored the use of DESs in ACM reactions for the synthesis of 2H-chromene derivatives (Scheme 13). The orto-progylated salicylaldehyde served as the starting material for the metal-free ACM reaction under mild reaction conditions [76]. Many literature-known DESs, such as ChCl:Urea (1:2), ChCl:Glycerol (1:2), ChCl:Ethylene glycol (1:2), and ChCl:*p*TSA (1:2), were tested as alternative catalysts for this metathesis, under different temperatures and reaction times. Only the mixtures containing *p*TSA provided the desired chromene in quantities higher than trace amounts. Of all the assessed reaction conditions, the best optimized yield (93%) was obtained using ChCl:*p*TSA (1:2) at 45–50 °C, with a reaction time of 8 h. Increasing the reaction time to 24 h slightly decreased the yield by 1%.

The role of both ChCl and *p*TSA components in the catalysis were individually assessed through performing the reaction with the aid of acetonitrile as solvent. While the experiment with ChCl in ACN did not provide any product, the *p*TSA counterpart produced the desired chromene with a 33% yield, making it possible to deduce the importance of the latter component’s acidic character. Based on this information, the authors proposed a mechanism following the hypothesis that the oxophilicity of the acidic DES on the carbonyl group of the starting molecule promotes the desired cyclisation. Furthermore, by conducting this process in the dark and utilizing radical traps, the authors deemed all hypotheses of a radical pathway as inviable.

After the optimization, different derivatives were synthetized via this method, resulting in moderate to high yields, except for the derivatives with bulky substituents which impose a level of steric hindrance. Of all the fourteen variations, the only situation where no product formation was observed was when the starting molecule contained a terminal alkyne substituent.

The recyclability of the DESs was also investigated. After the synthetic process, each derivative was extracted from the DES with ethyl acetate and the DES was recycled up to five times, with a slight reduction in the yield (2% to 5%) between each cycle.

Overall, this methodology showed how useful DESs can be in ACM under metal-free conditions. Moreover, mild reaction conditions enable the reactions affording many 2H-chromene derivatives that can later be used as intermediates for the preparation of pharmaceuticals or be used themselves as bioactive molecules.

#### 2.2.2. Multicomponent Reactions

The Biginelli reaction was first reported in 1893 and the interest on the main product, dihydropyrimidinones (DHPM), increased because they are extremely useful pharmaceuticals, since they are calcium channel blockers, antihypertensive agents, and alpha-1-α-antagonists [77]. This reaction is a multicomponent reaction between an aldehyde, a urea derivative, and an acetoacetate. The first step consists of a Mannich reaction, i.e., a β-amino-carbonyl compound, also known as a Mannich base. The final step, a condensation reaction, results in the Biginelli product. The list of Biginelli reactions reported in the literature is endless, and the number of reports of using DESs in Biginelli reactions is also increasing significantly.

**Scheme 14:**
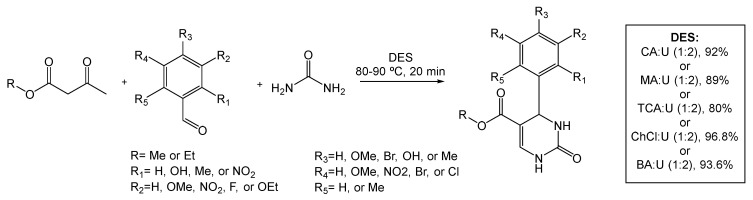
Biginelli reaction for the preparation of dihydropyrimidinones (DHPM) in DESs as alternative solvents.

Khan and colleagues [78] reported the synthesis of novel DHPM structures under sustainable conditions, and further test their inhibitory potential against cholinesterases (AChE and BChE) and monoamine oxidases A and B (MAO A and MAO B), common drug targets studied for the treatment of Alzheimer’s and Parkinson’s disease, depression, and anxiety. In this report, the Biginelli reaction was carried out with the aid of five different DES mixtures to prepare a total of 28 dihydropyrimidinones, which vary in substituent combinations (Scheme 14).

One of the substrates for the preparation of DHPMs is urea or urea derivatives. In the work proposed by Khan, by using DESs containing urea as one its components, the need of using another portion of urea as a reagent in the reaction mixture is conveniently avoided. Therefore, the five DESs prepared, all containing urea acting as both the hydrogen bond donor component and as the reagent, were first tested in a model reaction using benzaldehyde and ethyl acetoacetate as the starting materials. The desired product was obtained from the reaction with each low melting mixture, and was analyzed by 1H and 13C NMR.

The ideal reaction conditions were determined for each DES. Citric acid:urea (CA:U, 92% yield), malonic acid:urea (MA:U, 89% yield), trichloroacetic acid:urea (TCA:U, 80% yield), choline chloride:urea (ChCl:U, 96.8% yield), and benzoic acid:urea (BA:U, 93.6% yield), all in a 1:2 ratio, with a reported melting temperature ranging from 70 °C to 90 °C, and running times of 20–40 min. From the results, the authors concluded that, due to the maximum amount of yield in only 20 min and with mild temperatures not exceeding 80–90 °C, the eutectic system ChCl:U (1:2) afforded the best results.

The reaction time and melting temperature were also studied, via 3D response surface, as parameters to predict the yield of the model Biginelli reaction product. The results indicated that the temperature range of 80–95 °C allows for a >90% reaction yield and that the product could be obtained in a 20 min reaction time. After the referred optimization of reaction conditions, a variety of functionalized DHPMs were synthetized and their potential to inhibit MAO A and B was assessed. From the 28 DHMP derivatives, 5 were able to inhibit MAO A and 4 inhibited MAO B. In terms of AChE and BChE activity, 17 compounds presented AChE inhibition properties, and no compounds were able to inhibit BChE.

**Scheme 15:**
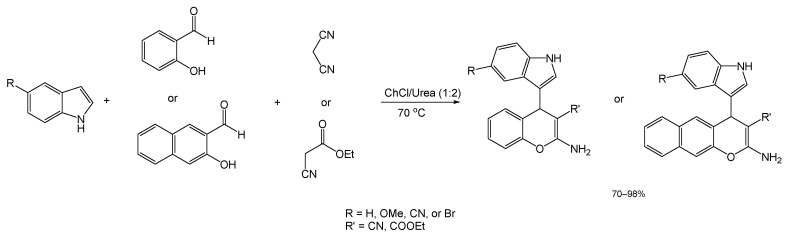
General reaction scheme and conditions for the preparation of indole-centered 4H-chromenes.

Another recent article describes the results of the search for an efficient DES for the preparation of indolyl chromenes with biological activity through multicomponent reaction. In this study, reported by Alvi et al. a model reaction between indole, salicylaldehyde, and malononitrile was used to screen several DES mixtures [79]. Of the DESs tested, ChCl/Urea (1:2), at a reaction temperature of 70 °C, rendered the best results, which translated to a yield of 98% and a reaction time of 30 min (Scheme 15).

Assembling the desired indole-based chromene derivatives was then achieved by applying the optimized reaction conditions to the same process but with various 5-substituted indole species. This process resulted in 4 different synthesized derivatives with yields from 68 to 98%.

Aiming for a wider diversity of compounds, the authors performed a variation of this process (under identical conditions), in which the malononitrile reagent was switched for ethyl cyanoacetate (Scheme 15), a process that also brought about the expected products in great yields. The researchers then created another two variations for the first reaction with malononitrile, via changing the salicylaldehyde for either 5-bromo-2-hydroxybenzaldehyde or 2-hydroxy-1-naphtaldehyde. The resulting product from the former variation underwent a Suzuki–Miyaura cross-coupling reaction with boronic acids (not performed under green conditions) to explore the possibility of generating Suzuki adducts (with the potential for medicinal activity) from the structures synthesized using a DES catalyst.

In this study, no significant decline in yield after four cycles of DES use was observed, thus implying the effective recyclability of it as both a reaction medium and a catalyst. The green properties and easy workup procedures coupled with these low melting mixtures highly suggests that this method is advantageous in comparison to the employment of traditional catalysts and solvents.

Other compounds that can be obtained through a multicomponent reaction are the dihydropyrano[c]chromene and tetrahydrobenzo[b]pyran derivatives, which are found in the form of naturally occurring compounds with diverse biological activities, including anti-diabetic, antimicrobial, antibacterial, antioxidant, anti-proliferative, anti-HIV, anti-tumor, anti-fungal, and anti-cancer properties.

**Scheme 16:**
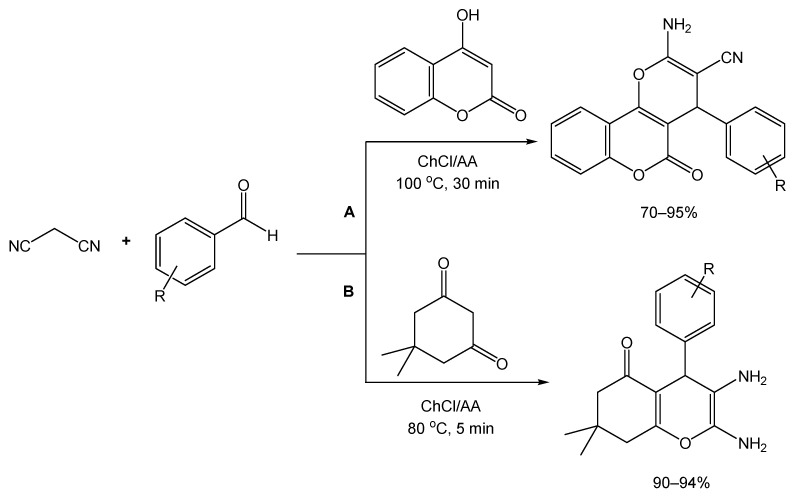
Synthetic approach of (**A**) dihydropyrano[c]chromene and (**B**) tetrahydrobenzo[b]pyran derivatives, using ChCl/AA as reaction medium and catalyst.

From a synthetic point of view, these derivatives can be obtained from a three-component reaction between an aldehyde, malononitrile, and a β-dicarbonyl compound, a reaction which has been improved before via the use of different catalysts, but presenting drawbacks such as high cost, environmental impact, and long reaction times. To overcome this, Valipour and colleagues [80] were inspired by other reports where DESs were used for the synthesis of these types of derivatives, and they followed the same approach via preparing a DES composed of ChCl/ascorbic acid (AA) (2:1), and conducting a preliminary study with a model reaction involving 4-chlorobenzaldehyde, malononitrile, and 4-hydroxycoumarin (Scheme 16A). By varying temperature, reaction time, and catalyst amount, conducting the reaction at 100 °C and using the DES was considered the best set of conditions for the synthesis of the desired dihydropyrano[c]chromene derivatives, resulting in a yield of 90% after 35 min. Using these conditions to a variety of substituted aldehydes with electron-donating and withdrawing groups, it was determined that the use of aromatic aldehydes containing EWGs afforded better yields and reaction times when compared to the EDG counterparts.

For the preparation of tetrahydrobenzo[b]pyran derivatives, dimedone was used instead of 4-hydroxycoumarin in the three-component reaction. For this substrate, 80 °C and 5 min of reaction time was sufficient to achieve a 97% yield. As opposed to the first set of derivatives, yields for the preparation of tetrahydrobenzo[b]pyrans whose starting materials contained either EWG or EDG were both reported as excellent (Scheme 16B).

Interestingly, the use of aliphatic aldehydes as starting materials was reported not to render the desired compounds, leading instead to an intermediate product which, under the tested conditions, does not proceed well in further reactions with 4-hydroxycoumarin or dimedone.

After investigating the recyclability of the catalyst using one of the 4H-pyran synthesis reactions as a proof-of-concept test, the authors reported the ability to re-utilize the DES catalyst for up to three cycles, with only a slight decrease in activity.

Finally, a comparison study was performed to evaluate the catalytic performance of ChCl/AA NADES against the previously reported methods, highlighting similar yield rates for the reference reactions of both types and a lower reaction time for the tetrahydrobenzo[b]pyran reference reaction.

The three examples presented herein showed how powerful DESs can be in multicomponent reactions and the major advantages that come from their use.

#### 2.2.3. Cyclization Reactions

In organic synthesis, there are a wide range of cyclization reactions, including the Diels–Alder cyclization, the Nazarov cyclization, Lewis-acid-promoted cyclization, and the Paal–Knorr cyclization, among others. Cyclization is a key reaction in medicinal chemistry, since most active molecules possess a cyclic ring in their structure. Examples of cyclic compounds are the quinazolinones, which are present in more than 150 naturally occurring alkaloids and many marketed drugs with anti-microbial, anticonvulsant, sedative, hypotensive, antidepressant, and anti-inflammatory properties, among others [81]. Some quinazolinones, such as methaqualone (Quaalude), mebroqualone, and mecloqualone (Casfen) have sedative, hypnotic, and anxiolytic properties, being used in insomnia treatment.

Although many reports are found in the literature for the preparation of quinazolinones using a variety of catalysts, reagents, and reactions conditions [82,83,84,85], the use of DESs is quite limited [86,87].

**Scheme 17:**
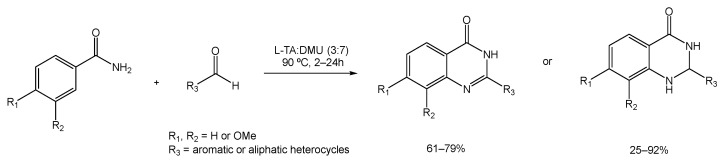
Dihydroquinazolinone synthetic strategy using L-TA:DMU as an alternative solvent.

Ghosh et al. studied the cyclization reaction between anthranilamides and aldehydes (either aromatic, aliphatic, or heterocyclic) to provide dihydroquinazolinone, which is later converted to quinazolinone under aerobic conditions [88]. The reaction of anthranilamide and orto-tolualdehyde was used as a model to test a group of mixtures that includes citric acid:DMU, D-(−)-fructose:DMU, L-(+)-tartaric acid:DMU, and mannose:DMU:NH_4_Cl, out of which the ratio of L-(+)-tartaric acid:DMU (3:7) was deemed the most effective yield at 90 °C (Scheme 17).

The efficiency of conversion from the dihydroquinazolinone form to quinazolinone varied depending on whether the anthranilamides and aldehydes used contained electron-withdrawing or electron-donating moieties. The presence of electron-withdrawing groups resulted in the desired dihydro derivative within 2 h, but it did not convert it to the corresponding quinazolinone after 24 h. For the aromatic and aliphatic anthranilamides and aldehydes, the cyclized product was obtained within 6 to 15 h in good yields, while the use of benzaldehyde afforded both forms of the product after 24 h.

Bouchardatine, a naturally occurring β-indoloquinazoline alkaloid, with anti-obesity properties has been used, in the same report, as a model for this cyclization using DESs as alternative solvents. The authors used anthranilamide and indole-2-carboxaldehyde. The reaction afforded the cyclized compound in an 81% yield after 8 h, which was then formylated under non-DES conditions, using DMF and POCl3 at 0 °C to obtain the desired bouchardatine after 24 h.

Schizocommunin, another natural alkaloid, with cytotoxic activity against murine lymphoma cells, was prepared. According to the authors, the only synthetic approach for this molecule comprises the transformation of the commercially available 2-methyl-4(3H)-quinazolinone. However, the same product can be obtained using a greener approach through the treatment of anthranilamide with 3 equivalents of acetaldehyde in L-(+)-tartaric acid:DMU (3:7) at 90 °C, which provided the dihydro form in a 79% yield after 2 h of reaction time. Further treatment with DDQ in dry DCM at room temperature produced the compound in a reaction time of 30 min. The final conversion to schizocommunin was carried out via a reaction reported by Nishida et al. [88].

**Scheme 18:**
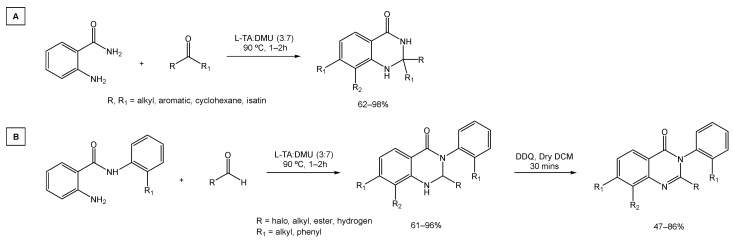
Synthesis strategy for the preparation of (**A**) 2,2′-disubstituted quinazolinones and (**B**) 2,3-disubstituted quinazolinones.

To further show the versatility of this green approach, Ghosh and co-workers investigated the synthesis of 2,2′- and 2,3- disubstituted quinazolinones (Scheme 18A and 18B, respectively). These processes produced a variety of desired compounds in good to excellent yields.

Another type of cyclic scaffold commonly found in nature is the benzimidazole ring. It is frequently named “privileged nucleus” because it is present in many essential molecules, such as vitamin B12, which is needed in the production of red blood cells and DNA.

The clinical application of benzimidazole-containing drugs includes the fungicide and antiparasitic thiabendazole, the antihistamine clemizole, the anti-ulcerative omeprazole, the anti-hypertensive telmisartan, the antiviral Hoechst 33342, and the anticancer bendastumide [89]. Over the years, several methods were studied for the preparation of benzimidazole derivatives, involving the reaction between o-phenylenediamines and carboxylic acids or derivatives [90].

**Scheme 19:**
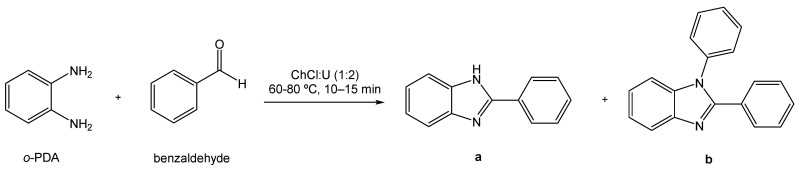
Synthesis of benzimidazole derivatives using ChCl:U as the solvent.

Following the trend of using greener processes and greener raw materials, alternative solvents have been investigated by Di Gioia et al. through using the eutectic system ChCl:U (1:2) as the reaction medium in a model reaction between o-phenylenediamine (o-PDA) and benzaldehyde that produced two distinct products (Scheme 19) [91]. The complete conversion of reagent to products was observed; however, the ratio of each product obtained in relation to the other depends on the reaction conditions and the molar ratio of the reagents. For example, when a ratio of 1:1 of o-PDA and benzaldehyde was used, at either 60 or 80 °C, 2-substituted benzimidazole derivative (a) was the major product. Nonetheless, if the ratio is 1:2, at both temperatures, the main product is the 1,2-disubstituted benzimidazole derivative (b).

**Scheme 20:**
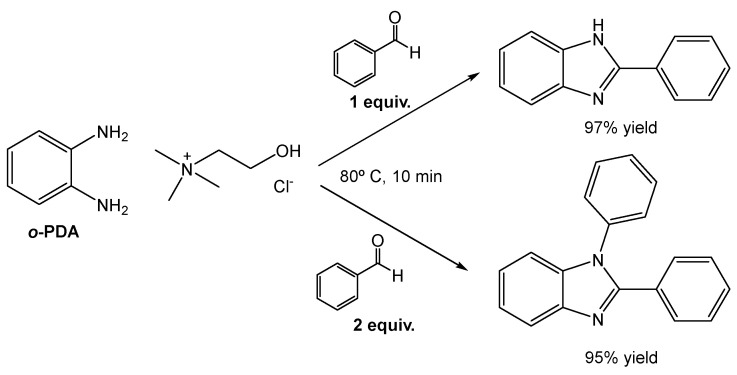
Selective synthesis of benzimidazole derivatives using a ChCl:o-PDA eutectic system with solvent and reagent dual function.

The authors then prepared a ChCl:*o*-PDA based DES, to include a component of the reaction medium as a reactant (Scheme 20). The preparation of this DES and its subsequent employment in the model reaction furnished similar results with great yields. But, most importantly, the use of this new DES allowed them to selectively obtain the mono- or the di-substituted derivative, depending on the aldehyde equivalents used, without affecting the reaction yield (97% and 95% for each product).

Most advantages reported for DESs for similar procedures are also observed in this work, namely recyclability, easy workup, mild reaction conditions, and, in this case, a scale-up test in the scale of 20 mol which had a positive outcome of 93% isolated yield.

#### 2.2.4. Condensation Reactions

A chemical reaction between two molecules that form a single molecule, usually with the loss of water (or another small molecule), is called a condensation reaction. The most common condensation reactions are the aldol condensation and the Knoevenagel condensation, in which water is lost as a by-product. While aldol condensation involved the reaction between two carbonyl moieties (aldehydes or ketones) to form a β-hydroxyaldehyde or β-hydroxyketone, the Knoevenagel condensation is the nucleophilic addition of an active hydrogen compound to a carbonyl group, followed by a dehydration reaction. But there are other similar reactions such as Claisen condensation and Dieckman condensation, that produce alcohols as by-products [92].

Rhodanine-based compounds are mostly prepared via Knoevenagel condensation. These molecules have been widely reported for their biological activity and their relevance in drug discovery, especially in the field of type 2 diabetes. There are many reports on their preparation using traditional solvents [93], but also on alternative methods that may include microwave technology [94] and green catalysts [95].

A recent study carried out by Hesse [96] reported the preparation of rhodanine derivatives via Knoevenagel condensation using the eutectic system ChCl:U (1:2). The author was inspired by others’ work, where L-proline-based DESs were used as reaction media in Knoevenagel condensations for the synthesis of other classes of compounds. In this regard, the author explored the hypothesis of optimization of rhodanine synthesis through using the system Pro:Gly (1:2), which is relatively cheap to produce, and reported it as recyclable.

**Scheme 21:**
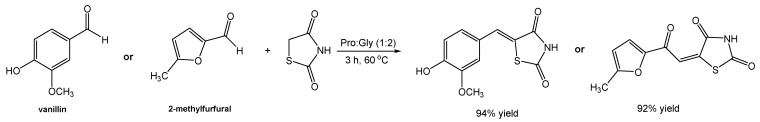
Synthetic approach for the preparation of rhodanine derivatives using Pro:Gly (1:2) as the solvent.

Initially, a model reaction between vanillin and rhodanine in Pro:Gly (1:2) was studied (Scheme 21), at room temperature, affording the desired compound in a 57% yield, which was similar to the one reported by other authors but at 90 °C and using the eutectic system ChCl:U (1:2) [97]. Interestingly, changing the eutectic system to Pro:Gly (1:2) and carrying out the reaction at 60 °C made it possible to reach a maximum yield of 94%.

When the authors replicated the reported reaction using the system ChCl:U (1:2) but at a lower temperature (60 °C), a yield of 20% was obtained. However, when adding a catalytic amount of proline to this reaction, a slight increase in the final yield was observed (30%), confirming the importance of proline in the success of the reaction. Nevertheless, the maximum efficiency was achieved when proline was part of the eutectic system. A possible explanation has been hypothesized by the author, in which an iminium pathway catalyzed by L-proline may be possible, or even an activation of carbonyl groups by the DES. A second reaction using 5-methylfurfural as the substrate (alternative to vanillin), lead to a similar result of 92% yield after a reaction in Pro:Gly (1:2), as opposed to 20% in ChCl:U (1:2) and 75% in ChCl:U (1:2) containing a catalytic amount of L-proline. Later, the optimized reaction conditions were applied to other aldehydes where the final rhodanine derivatives were obtained with good to great yields (63–92%) in comparison to values found in the literature.

Chalcones are another class of compounds obtained via condensation reactions, more specifically by Claisen–Schmidt condensation [98,99]. Chalcones are widely spread in nature since they derived from flavonoids, and have been proven to be excellent bioactive molecules with a variety of pharmacological properties, especially antioxidant and anti-inflammatory.

**Scheme 22:**
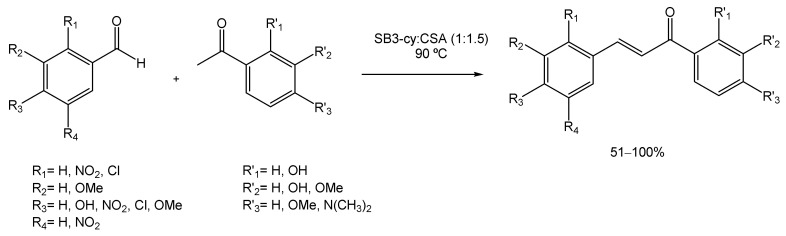
Chalcone synthesis via Claisen–Schmidt condensation, using DESs as alternative solvents.

Many strategies for their preparation have been developed over the years, from solvent-free [100] to ionic liquids [101]. But Cardellini et al. investigated the applicability of DESs in these reactions [102]. Therefore, the authors prepared a new DES composed of 3-(cyclohexyldimethylammonio)propane-1-sulfonate (SB3-Cy) and (1S)-(+)-10-camphorsulfonic acid (SB3-Cy: CSA) which was then used as both the reaction medium and the catalyst. To screen the use of the SB3-cy:CSA DES and to optimize the reaction conditions, the authors mixed benzaldehyde and acetophenone at 90 °C and allowed the reaction to complete (Scheme 22). The substrates were always used in a 1:1 ratio to minimize waste production. To achieve the maximum reaction yield, different temperatures were tested; however, the results showed that higher temperatures (140 °C) gave the best results. Therefore, to make it greener, the temperature was maintained at 90 °C and the reaction time increased up to 16 h, allowing the authors to achieve the best results. Afterwards, benzaldehyde was combined with mono- and bi-substituted acetophenones, and acetophenone was combined with mono- and bi-substituted benzaldehydes (Scheme 22) and in all cases, good to excellent yields were obtained (from 51% to quantitative). The DES’s recyclability was also studied, and after four cycles the yield did not suffer a significant reduction.

This is another example where DESs can play a powerful role in drug discovery. By using them, these scaffolds can be easily prepared and used either as they are or as building blocks for other relevant molecules.

#### 2.2.5. Total Synthesis

Although many reports found in the literature present a single reaction using DESs as alternative solvents, some authors reported the total synthesis, consisting of several steps, of different bioactive compounds using DESs as solvents.

For example, Piemontese and co-workers [103] reported the total synthesis of PZ1, a new multi-target ligand and AchE inhibitor for Alzheimer’s disease treatment. PZ1 belongs to the benzimidazole family, which has been widely explored for the preparation of bioactive molecules, such as Telmisartan (antihypertensive agent), Maribavir (antiviral drug), omeprazole (anti-inflammatory and antiulcer agent), and others.

**Scheme 23:**
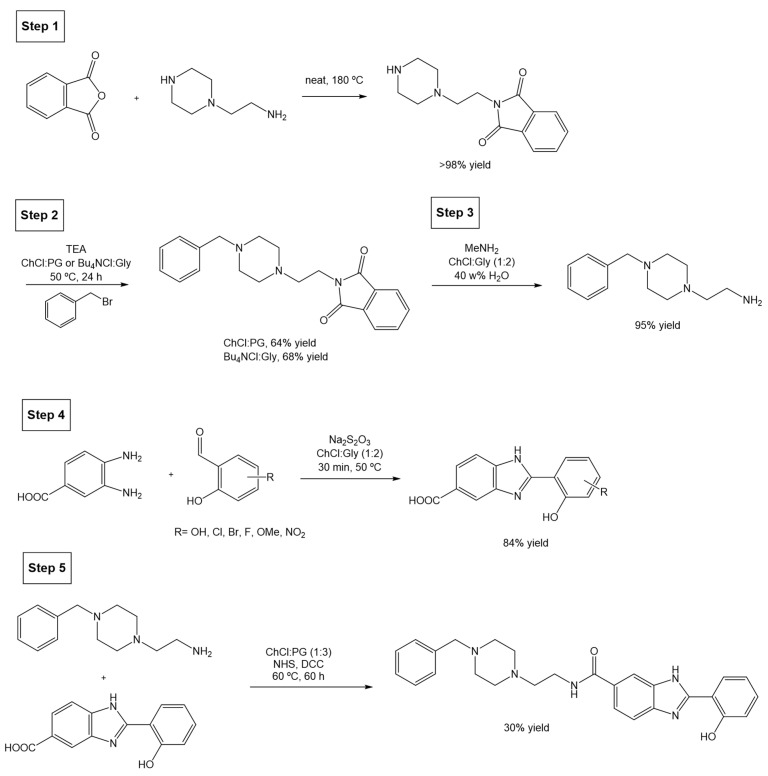
Total synthesis of PZ1 compound using DESs as alternative solvents.

The original total synthesis of PZ1 involves several steps where toxic reagents such as acetonitrile (ACN), *N,N*-dimethylacetamide (DMA), *N,N*-dimethylformamide (DMF), and the carcinogenic hydrazine hydrate are used. Inspired by the green chemistry principles, the authors redesigned the whole synthesis and included DESs as solvents (Scheme 23).

The first step of the synthetic pathway involves a solventless reaction with a quantitative yield, hence not posing a problem; but the second reaction (step 2), a benzylation on the piperazine moiety (secondary amine) of the starting molecule, originally uses triethylamine (TEA) and K_2_CO_3,_ and is carried out in acetonitrile at 50 °C for 3 h. Although the authors attempted several different reaction conditions, using different DESs, base combinations, temperatures, and reaction times, the most promising strategy switches ACN for a reaction medium composed of either ChCl:PG (1:3) or Bu_4_NCl:Gly (1:4) at 50 °C, using TEA as a base, and obtaining a yield of 64% and 68%, respectively, after 24 h.

The next optimized step is the deprotection of the phthalimide moiety (step 3), originally carried out using hydrazine hydrate in refluxed EtOH, with a 95% reaction yield. Changing these conditions for methylamine (MeNH_2_) in ChCl:Gly (1:2) and 40 w% water provided the deprotected amine with a similar yield of 95%. Alternatively, when using the eutectic mixture ChCl:PG (1:3) in 40 w% a significant reduction in the reaction yield (45%) was observed.

In parallel, the synthesis of 2-hydroxyphenylbenzimidazole, which is later coupled with the previous amine, was also studied. The first step (step 4) consists of a cyclodehydration reaction between 3,4-diamoniobenzoic acid and salicylaldehyde. Originally, the method is carried out in the presence of the toxic DMA, at a high temperature (100 °C) and with a 12 h reaction time. The alternative reaction was optimized to a 30 min reaction at a much milder temperature (50 °C) in the presence of a DES as solvent, using Na_2_S_2_O_5_ as the oxidant. Different DESs were tested, namely ChCl:Gly (1:2), ChCl:U (1:2), and ChCl:LA (1:2), but the first was found to obtain the best yield. An interesting and highly relevant outcome from this optimization was that the cyclodehydration product was found to precipitate from the ChCl/gly mixture after dilution in water, allowing for a simple isolation. Overall, this synthetic step improves not only the sustainability of the process, but also the yield (67% in the classical reaction compared to 84%), reaction time (12 h to 30 min), and heating temperature needed (100 °C to 50 °C).

The final coupling step (Scheme 23, step 5) between the amine from step 3 and the carboxylic acid from step 4, is usually achieved using *N,N*’-dicyclohexylcarbodiimide (DCC) and *N*-hydroxy-succinimide (NHS) in DMF as solvent, at 25 °C for 60 h and with a final yield of 21%. Using the greener approach, i.e., using the eutectic system ChCl:PG (1:3), a higher yield (30%) is achieved after running for 60 h at 60 °C.

In a different study, Quivelli et al. reported the first sustainable procedure for the preparation of Thenfadil, a first-generation antihistamine drug used in the treatment of allergies such as urticaria, hay fever, rhinitis, and asthma. It is a drug which garners attention due to its unusually high activity and low toxicity when compared to methapyrilene, another drug used for the treatment of allergies [104].

According to the authors, the original method, developed and patented in 1951, for the synthesis of Thenfadil, is still widely used for its on-demand production at EUR 325/50 mg. This method involves the reaction between a 2-(dimethylminoethylamino)-substituted pyridine and 3-bromomethylthiophene in refluxing toluene in the presence of sodium amide (NaNH_2_).

**Scheme 24:**
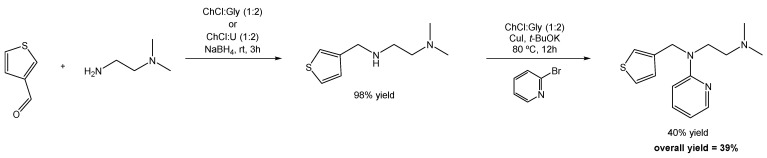
Total synthesis of Thenfadil using DESs as alternative solvents.

Inspired by the climate crisis and the need for change in terms of the chemicals/solvents used in the production of pharmaceuticals, Quivelli and co-workers redesigned the synthesis of Thenfadil, and developed an optimized and sustainable one-pot, two-step synthesis combining a reductive amination with an Ulmann-type C-N coupling reaction, using DESs as the reaction media in both cases (Scheme 24). This first step of reductive amination starts with the addition of the diamine to the 3-thiophenecarboxaldehyde in ChCl:Gly or ChCl:U (1:2), at room temperature and under air, in the presence of NaBH_4_. The product was obtained in a 98% yield.

The second step is a C-N Ulmann coupling reaction in which the previous compound reacts with bromopyridine, copper iodide (CuI), and potassium *tert*-butoxide (*t*-BuOK) as the base, suspended in ChCl:Gly (1:2 mol) under air and stirring at 80 °C. The final product is obtained in a 40% yield.

The overall yield for this new synthesis of Thenfadil is 39%, while the traditional method reported by Campaigne and LeSuer is reported to be 30% [104]. Not only was it possible to slightly increase the overall yield of the reaction, but the new method also uses much smoother reaction conditions and is performed under aerobic conditions, contrarily to the traditional method.

To quantify and validate this method’s improvement and sustainability in comparison to the original work from Campaigne and Bourgeois, some green metrics were taken into account such as atom economy (AE), reaction mass efficiency (RME), optimum efficiency (OE), effective mass yield (EM), mass intensity (MI), and process mass intensity (PMI), but also the metrics of space time yield (STY), renewables intensity (RI), and renewables percentage (RP). Most of these parameters supported the advantage of using DESs in this specific process, such as RME which increased from 4.5 to 14.1% or EM which increased from 0.6 to 6.7%, among others.

Furthermore, for scale-up purposes, the authors were able to increase the amount of substrate up to 50 g and DESs up to 500 g without any impact on the yield. Additionally, the robustness of the new method was tested through synthesizing Thenfadil analogs, namely, tripelennamine, methaphenilene, and thonzylamine (with overall yields of 39%, 44%, and 39%, respectively).

Finally, and just as importantly, the production cost of Thenfadil using this methodology was analyzed. As mentioned before, currently this drug is produced at EUR 325/50 mg. Using this new strategy, it would be possible to produce 51.6 mg of Thenfadil with a production cost of EUR 0.365, which is tremendously lower.

The examples presented in this last section clearly show the great impact that DESs may have in the industrial production of APIs, while sparing, at the same time, the environment to hazardous chemicals and solvents.

Furthermore, the choice of the solvent and/or catalyst for a specific reaction must be carefully evaluated, considering the green chemistry metrics. We believe that to move DESs towards the industry, researchers must start to include sustainability studies and green chemistry metrics in their work to prove the benefits of these new solvents, especially in the field of pharmaceutical sciences since this is one of the greatest contributors for the increase in carbon footprint.

## 3. Conclusions

In this review, we have revised the latest application of deep eutectic systems (DESs) as reaction media in the preparation of building blocks and intermediates for the synthesis of APIs, or of APIs themselves. We showed that DESs can be easily used in either biocatalytic processes or organic synthesis allowing for milder and faster reaction conditions, and easier separation/purification processes. Furthermore, reports showed that DESs can lead to a much more sustainable and cheaper production of APIs, mainly due to the proved recyclability and green metrics. Nevertheless, researchers need to pay special attention to the separation process and recyclability of the DESs, since several things can happen during the processes, namely, evaporation of one of the DES components and separation of the DES components during extraction steps, among others. To the extent of our knowledge, and according to the reports summarized herein, a full characterization of DESs post-reactions was never performed. We do believe that this should be considered in future studies, to ensure that during recycling steps the media is, in fact, of the original DES composition.

As demonstrated by some studies presented herein, the use of DESs as solvents but also as reagents presents major advantages, and this should be further explored in future works. Also, although many reactions have not yet been extensively studied using DESs as alternative solvents, we believe that soon more research will be carried out in this field and hopefully it will pave the way for pharmaceutical companies to start to incorporate DESs in their industrial production of APIs.

## Data Availability

Not applicable.

## Nomenclature

Ala	alanine
Bet	betaine
ChCl	choline chloride
EACl	ethyl ammonium chloride
EG	ethylene glycol
PG	propylene glycol
Glu	glucose
Gly	glycerol
Sor	sorbitol
AchE	acetylcholinesterase
BchE	butirylcholinesterase
LA	lactic acid
DMU	dimethylurea
TCA	trichloroacetic acid
MA	malonic acid
CA	citric acid
BA	benzoic acid
U	urea
L-TA	L-tartaric acid
